# Comparison of the Levels of Hematological Parameters at Rest and after Maximum Exercise between Physically Active People with Spinal Cord Injury and Able-Bodied People

**DOI:** 10.3390/ijerph182312323

**Published:** 2021-11-24

**Authors:** Łukasz Szymczak, Tomasz Podgórski, Katarzyna Domaszewska

**Affiliations:** 1 Faculty of Health Sciences, Calisia University, 62-800 Kalisz, Poland; l.szymczak@akademiakaliska.edu.pl; 2 Department of Physiology and Biochemistry, Poznan University of Physical Education, 61-871 Poznan, Poland; podgorski@awf.poznan.pl

**Keywords:** spinal cord injury, hematological parameters, peak oxygen uptake

## Abstract

The aim of the study was to reveal the difference in the hematological reaction to the applied exercise-induced workload between the able-bodied and physically active people with cervical spinal cord injury. The study covered 11 males with spinal cord injury and 11 able-bodied persons. An incremental stress test was carried out until the maximum individual workloads were achieved. The peak oxygen uptake was measured with the use of the ergospirometric method. Venous blood test results at rest and after finishing the maximal exercise showed hemoglobin (Hb) concentration, hematocrit (HCT) value, erythrocytes (RBC), leukocytes (WBC) and platelets (PLT) counts as well as the relative percentage of granulocytes (GRA), lymphocytes (LYM), and monocytes (MON). RBC, HCT as well as Hb and PLT among people with the injury were statistically lower (*p* < 0.001) large effect size, than in the control group. Statistically significant difference between the test and control group, subjected to the maximal exercise stress test, was observed in the exercise induced change of the PLT [*p* < 0.001, (ES: 2.631)] WBC [*p* < 0.05, (ES: 1.429)] and the percentage of LYM and GRA [*p* < 0.05, (ES: 1.447) for LYM and (ES: 1.332) for GRA] between both groups, subjected to the maximal cardiac stress test on the manual cycloergometer. The analysis of the obtained results indicates that people with spinal cord injury are much more vulnerable to the occurrence of microcytic anemia compared to able-bodied people. The after-exercise percentage shift of selected subpopulations of leukocytes in both groups indicates a delayed post-exercise recovery among people with spinal cord injury.

## 1. Introduction

Hematological disorders, particularly anemia, are a very common complication of spinal cord injury in the acute phase, i.e., upon the occurrence of the injury, even when there is no blood loss (gastrointestinal bleeding, hemorrhage). The erythropoiesis rate is regulated by such factors as hormones, nervous system, and hypoxia. Hormones regulating red blood cell production rate include erythropoietin, thyroid hormones, growth hormone, and cortisol [1]. The influence of the nervous system, in turn, is related to the regulation of adrenaline and noradrenaline secretion by the adrenal medulla. These hormones work in a similar way and cause a change in the contractility of blood vessels, thereby reducing blood flow through the kidneys. It leads to hypoxia-induced increase in the erythropoietin production. Furthermore, among people with spinal cord injury, a reduction in the concentration of adrenaline circulating in the blood can be observed, which is caused by the disconnection of the adrenal sympathetic nervous system [2,3,4]. Understanding the etiology of anemia in this group of patients is a prerequisite to prevent its consequences. The analysis of hematological parameters of patients with spinal cord injury indicates the occurrence of different types of normocytic and microcytic anemia. A reduced level of red blood cells count does not significantly endanger patients’ life but can contribute to developing secondary complications related to tissue hypoxia [5,6].

Rehabilitation of people with spinal cord injury is aimed at improving their functional capacities and increasing their injury induced decreased exercise tolerance level. Regular physical activity may sharpen their wheelchair skills and allows to maintain muscle mass. Additionally, it limits the occurrence of adverse circulatory and respiratory changes. Physical activity is also conducive to the prevention of osteoporosis, diabetes, or neoplastic diseases. Patients with cervical spinal cord injuries who undertake physical activity are less likely to suffer from bedsores, muscle contractures, muscle tension disorders, and orthostatic blood pressure decline. Moreover, regular exercises limit the occurrence of adverse hematological and biochemical changes in the blood [7,8,9,10,11].

The volume of plasma changes during a physiological reaction to the exercise-induced workload, which leads to hemoconcentration or hemodilution. Their occurrence depends on the intensity and duration of the exercise as well as the physical endurance capacity of the person taking the effort. Hemoconcentration increases blood viscosity and occurs during short term effort with high intensity. Long term effort leads to an increase of oncotic blood pressure and its dilution [12,13]. Along with the intensification of the physical activity, increasing blood lactate concentration and a series of hormones capable of modulation within leukocytes is released. Exercise-induced leukocytosis is not a consequence of changes in the volume of plasma but results from the increased cell traffic from bone marrow to blood, demargination from blood vessel walls, and decreased exit to tissues. Animal experiments suggest that signals from inflamed tissues are humoral, not nervous. Some of these humoral factors are adrenaline, noradrenaline, growth hormone (GH), and cortisol, as well as plasma granulocyte colony-stimulating factor (G-CSF) and interleukin-6 (IL-6). The multiple stage process of leukocytosis is characterised not only by a change in the total white blood cell count, but also in the percentage of each of their fractions. At the lymphocytic stage, during moderate effort the lymphocyte count increases to 55% and neutrophil granulocyte count decreases. The total count of leukocytes in the blood does not change. Intensification of the effort increases the neutrophil granulocyte count to 78% and decreases the lymphocyte count below the values at rest. The total leukocytes count may increase even to 12 × 10^9^ L^−1^. Very intense effort, though not exceeding the possibilities of the body, leads to the intoxicative phase. The white blood cell count may increase even to 20 × 10^9^ L^−1^. Undertaken effort that significantly exceeds one’s performance capacities leads to leukopenia and a decrease in immune resistance. Most likely, this phenomenon results from metabolism disorders and insufficient capacity of protein synthesis in the changed conditions. Hormonal mechanisms responsible for the exercise-induced change in the leukocyte count also cause exercise-induced thrombocytosis [14,15,16].

The aim of the study was to answer the question whether there is a difference in the hematological reaction to the applied exercise-induced workload between the able-bodied and physically active people with cervical spinal cord injury.

## 2. Materials and Methods

The study was conducted according to the Declaration of Helsinki and the National Statement and Human Research Ethics Guidelines and approved by IRB (Institute for Research in Biomedicine) at the Poznan University of Medical Sciences (5 April 2012; Ethics Approval Number: 405/12). All participants in this study gave their written informed consent. The research covered 11 members of the Polish national wheelchair rugby team with spinal cord injury at the cervical level, ranging in age from 21 to 41 years (test group), and 11 able-bodied male students of the Poznan University of Physical Education Department (control group). These persons had an average level of physical endurance (Table 1) and had comparable training experience to the study group. The disabled with spinal cord injury subjected to the study constitute a homogeneous group in terms of age, body height, weight, and the injury level. All of them were tetraplegics with cervical spinal cord injury at levels C5–C7. Wheelchair rugby classification system, used to assess the subjects came from the ISMGF/ISMWSF (International Stoke Mandeville Games Federation/ International Stoke Mandeville Wheelchair Sport Federation) and American Spinal Injury Association (ASIA) Impairment Scale, medical classification system [17]. All the disabled research participants started training wheelchair rugby relatively early and gained considerable experience in the sport. The anthropometric characteristics of the groups as well as the degree of their spinal cord injury and training experience are presented in Table 2 and Table 3.

### 2.1. Assessment of Aerobic Fitness (Graded Exercise Test Protocol)

The exercise tests were conducted between 8:00 a.m. and noon in an air-conditioned laboratory, 2 h after consuming a light breakfast (one sandwich with butter and cheese; approx. 200 kcal). The subjects performed a cardiac stress test with increasing intensity on the REHAB TRAINER 881E arm cranking manual ergometer, manufactured by Monark Exercise AB (Vansbro, Sweden) and specially adjusted to functional abilities of research participants. The initial workload amounted to 10 Watts seconds (60 revolutions per minute RPM) was successively increased by 10 Watts every 3 min until the subjects achieved maximum in dividual workloads or refused to continue the effort. Peak oxygen uptake (peakVO_2_) and oxygen consumption per minute at the level of ventilatory threshold (VO_2_VT) using the “V-slope” method were measured in absolute and relative values with the German Jeager Oxycon Mobile gas analyser (Viasys Healthcare, Höchberg, Germany).

### 2.2. Preparation of Blood Samples for Analysis

Venous blood was taken twice from the ulnar veins, i.e., at rest (fasting blood; 6:00 a.m.) and 5 min after finishing the exercise using a S-Monovette syringe (Sarstedt, Nümbrecht, Germany) containing K_3_EDTA as an anticoagulant. Hemoglobin (Hb) concentration, hematocrit (HCT) value, total erythrocyte (RBC), leukocyte (WBC) and platelet (PLT) counts as well as the relative percentage of granulocytes (GRA), lymphocytes (LYM) and monocytes (MON) were measured immediately after blood collection and the samples were determined with the use of the MYTHIC 18^®^ hematology analyser (PZ Cormay SA, Łomianki, Poland).

### 2.3. Statistical Analysis

All the data are presented as mean (standard deviation). In order to calculate the significance of changes in the studied parameters, the Wilcoxon sign test of pairs was performed. The importance of differences between the test and control group was calculated with the Mann–Whitney U test. Correlation between the variables was calculated with the Spearman’s rank test. The level of statistical significance was set at *p* < 0.05. All results were statistically analysed using Dell Inc. (2016), Dell Statistica v.13. (Tulsa, OK, USA). Effect sizes [ES] were calculated as the difference between means divided by the pooled standard deviation. Using Cohen’s (1988) criteria, an effect size ≥ 0.20 and <0.50 was considered small, ≥0.50 and <0.80 medium, and ≥0.80 large [18].

## 3. Results

Table 4 presents morphological blood parameters as mean and standard deviation, measured at rest and after exercise in both groups. RBC, HCT as well as Hb and PLT counts among people with the injury were statistically lower (*p* < 0.001) large effect size, than in the control group. The analysis of the Mann–Whitney U test did not reveal any significant differences in the exercise-induced change in the red blood cell count, hemoglobin concentration, hematocrit value and the percentage of monocytes in the blood between able-bodied and disabled research participants. However, a statistically significant difference was observed in the exercise-induced change of platelet count [*p* < 0.001, (ES: 2.631)] white blood cell count [*p* < 0.05, (ES: 1.429)] and the percentage of lymphocytes and granulocytes in the blood [*p* < 0.05, (ES: 1.447) for lymphocytes and (ES: 1.332) for granulocytes] between both groups, subjected to the maximal cardiac stress test on the manual cycloergometer.

To assess physical endurance and exercise tolerance, research participants performed an exercise with increasing intensity on the manual ergometer. Table 1 presents average values and standard deviations of oxygen consumption (in absolute and relative values) obtained at peak workload and at the level of ventilatory threshold. The difference in each aerobic capacity parameter was statistically significant between the groups (*p* < 0.001). The subjects with spinal cord injury obtained about 50% lower results both at the level of threshold and peak workloads, compared to the able-bodied subjects within the same model of physical exercise workload (Table 1).

We observed, a statistically significant positive correlation between the spinal cord injury level and the exercise-induced change in the relative percentage of neutrocytes (*r* = 0.66, *p* < 0.05), the training experience and exercise induced increase of the total leukocyte count (*r* = −0.61, *p* < 0.05), the value of peakVO_2_ and the exercise induced decrease of the relative percentage of lymphocytes (*r* = −0.70, *p* < 0.05) and the change in the relative percentage of monocytes (*r* = −0.69; *p* < 0.05).

## 4. Discussion

The reduction of aerobic capacity of the people with cervical spinal cord injury is multifactorial. Due to the paralysis of lower limbs and partial paralysis of upper limbs, the activity of the muscle pump drops. In consequence, keeping venous blood return to the heart is insufficient. Reduced values of hemodynamic parameters and their exercise-induced course, different from the one of the able-bodied people, are caused by the lack of influence of the sympathetic system on the heart muscle, the state of blood vessels or secretory actions of endocrine glands. In the case of people with cervical spinal cord injury, the rest and exercise induced values of circulatory and respiratory parameters as well as the biochemical blood parameters differ from those of the able-bodied people or limbless amputees with maintained mechanisms of stimulation of the sympathetic nervous system [19,20,21,22,23]. Physical activity undertaken by people with spinal cord injury is of crucial importance in keeping the appropriate state of their physical capability and exercise tolerance. Hence, the oxygen efficiency parameters of the subjects from the test group were very high, taking into account the degree of their spinal cord injury. The results obtained by the disabled research participants in the in-house study of the peakVO_2_ value averaged at 16.82 ± 7.569 mL·kg^−1^·min^−1^. Moreover, 10 subjects from the test group reached individual threshold workload at the level of peakVO_2_ 79.93%. The intended threshold oxygen consumption averaged at 1046.00 ± 302.860 mL·min^−1^; whereas the relative value amounted to 14.94 ± 4.519 mL·kg^−1^·min^−1^. Average values of this parameter observed in the in-house study were considerably higher than those obtained by other researchers of this group of people and 50% lower compared to the able-bodied subjects. This result was statistically significant (*p* < 0.001). Dreisinger et al., while studying training tetraplegics, noted the values of oxygen consumption at the level of 780 mL·min^−1^, whereas Wicks et al. at the level of 13.8 mL·kg^−1^·min^−1^ [24,25]. Burkett et al. in their quite comprehensive research concerning both training and non-training people with spinal cord injury, noted the maximal oxygen consumption for physically inactive tetraplegics oscillating between 5.31–11.89 mL·kg^−1^·min^−1^. Undoubtedly, the clinical state after spinal cord injury, as well as the physical capability level, influence the values of the morphological parameters at rest and after exercise [26,27]. Huang et al. examined 28 people with cervical spinal cord injury between C3 and C7 vertebrae and revealed that several weeks after the injury, hematologic parameters of 9% of the subjects from the test group were lower than reference values. The changes concerned erythrocytes and reticulocytes counts, hematocrit value, hemoglobin, and iron concentration in the blood. Huang et al. demonstrated the occurrence of normocytic anemia among 71% of the people examined by him, whereas 14% of the subjects had microcytic anemia. These disorders influence the development of other secondary complications related to tissue hypoxia [5]. Perkash et al. and Davies et al. demonstrated the occurrence of a mild type of anemia in 52.3% out of 65 male patients with spinal cord injury subjected to the study. They did not confirm the correlation between age, duration of the injury or damage degree and the occurrence and type of anemia [28,29,30]. The in-house study demonstrated that people with cervical spinal cord injury had low values of the examined hematologic blood parameters at rest—below the range of physiological values (Table 4). RBC, HCT as well as Hb and PLT counts among people with the injury were statistically lower (*p* < 0.001) than in the control group. In case of WBC, no significant difference between the groups was observed. Only the percentage value of monocytes at rest was statistically higher in the control group (*p* < 0.05), and the maximal physical exercise caused the occurrence of poliglobulia phenomenon in each group. The exercise-induced blood density was higher among people with spinal cord injury, but the difference was not statistically significant. The exercise-induced thrombocytosis occurred in each of the tested groups, with lower statistically significant intensity among people with spinal cord injury (*p* < 0.001). Most likely, this difference results from the level of physical performance of the subjects and the degree of homeostatic imbalance of the body. The inflammatory reaction model described in the literature is a typical physiological reaction of the body to the workload in the exercise stress test [31]. In case of the people with the injury, the change of the specific fraction of white blood cells, along with a significant increase of the total leukocyte count, is the most similar to the model of changes of a neutrophil character. Furthermore we observed, a statistically significant positive correlation between the spinal cord injury level and the exercise induced change in the relative percentage of neutrocytes (*r* = 0.66, *p* < 0.05), the training experience and exercise induced increase of the total leukocyte count (*r* = −0.61, *p* < 0.05), the value of peak oxygen consumption and the exercise induced decrease of the relative percentage of lymphocytes (*r* = −0.70, *p* < 0.05) and the change in the relative percentage of monocytes (*r* = −0.69; *p* < 0.05). A statistically significant higher increase of the WBC count in the blood (*p* < 0.05) in reaction to the physical exercise of the group of able-bodied people was noted compared to the people with spinal cord injury, along with a statistically significant difference in the change in the percentage of lymphocytes between the groups (*p* < 0.05). The lack of exercise induced reduction in the percentage of lymphocytes among the able-bodied people was probably related to the lower exercise induced secretion of cortisol and growth hormone during the test effort [32]. One of the reasons responsible for the mechanisms of myogenic leukocytosis is, among others, a change in the concentration of neurohormonal factors (cortisol, catecholamines, growth hormone, endorphins, sex-steroids) and cytokines (TNF-α, IL-1, IL-6). A very similar model of reaction to exertion was described by Kouda et al., who revealed that the concentration of IL-6 and blood hematologic elements in plasma among able-bodied people increased significantly after finishing the exercise. However, they did not demonstrate that physical exercise causes such significant changes in case of the people with cervical spinal cord injury. They assumed that these differences might have been caused by muscular atrophy and sympathetic nervous system disorders among people with spinal cord injury [33]. The analysis of the obtained results revealed that people with spinal cord injury, despite high physical activity, run a much greater risk of microcytic anemias compared to able-bodied people. Furthermore, the after-exercise percentage shift of specific sub-populations of leukocytes in both groups (lymphocytes and granulocytes) indicates a later after-exercise regeneration of people with spinal cord injury.

## 5. Conclusions

The analysis of the obtained results indicates that people with spinal cord injury are much more vulnerable to the occurrence of microcytic anemia compared to able-bodied people. The after-exercise percentage shift of selected subpopulations of leukocytes in both groups indicates a delayed post-exercise recovery among people with spinal cord injury.

### Limitation of the Study

The study was conducted on physically active people with spinal cord injuries and healthy students. For a complete view of hematological changes under the influence of exercise, it would be necessary to perform tests on non-physically active males with damaged spinal cord. Unfortunately, for this study, the appropriate consent to conduct research on this group of people was not obtained.

## Figures and Tables

**Table 1:** Comparison of peak oxygen uptake and oxygen consumption obtained at the ventilatory threshold level.

Parameter	Test Group	Control Group	*p*-Value
VO_2 VT_ [mL·min^−1^]	1046.00 ± 302.860	1740.54 ± 258.058	0.0003
VO_2 VT_ [mL·kg^−1^·min^−1^]	14.94 ± 4.519	23.25 ± 3.245	0.0022
peakVO_2_ [mL·min^−1^]	1195.16 ± 482.197	2396.36 ± 327.269	0.0000
peakVO_2_ [mL·kg^−1^·min^−1^]	16.82 ± 7.569	32.14 ± 4.995	0.0001
peak Load [Watt]	43.18 ± 15.706	80.00 ± 10.445	0.0000
Exercise duration [min]	13.00 ± 4.632	24.00 ± 3.133	0.0000

Data are presented as mean ± SD, peakVO_2_ = peak oxygen uptake, VO_2 VT_ = oxygen uptake on VT. Bold indicates the statistically significant values.

**Table 2:** The anthropometric characteristics of the groups subjected to the study.

Parameter	Test Group	Control Group
Age [years]	34.18 ± 4.39	22.64 ± 2.38
Height [m]	1.81 ± 0.055	1.79 ± 0.073
Body mass [kg]	72.91 ± 11.71	75.36 ± 8.71
BMI [kg·m^−2^]	22.40 ± 4.38	23.25 ± 1.68

Data are expressed as mean ± SD; BMI = body mass index.

**Table 3:** Classification points, spinal cord injury level, American Spinal Injury Association (ASIA) Impairment Scale and training experience of the subjects with spinal cord injury.

No	Spinal Cord Injury Level	American Spinal Injury Association (ASIA) Impairment Scale	Classification Points	Time after the Injury [Years]	Training Experience [Years]	Time between the Injury and the Onset of Training [Years]
1	C6–C7	A	2	13	11	2
2	C5–C6	A	2	2	1	1
3	C6	A	2	6	3	3
4	C6–C7	A	2	5	3	2
5	C6–C7	A	2.5	17	13	4
6	C5–C6	A	2	13	8	5
7	C6–C7	A	2	12	6	6
8	C6–C7	A	2	9	7	2
9	C5	A	2	15	10	5
10	C5–C6	A	1	20	12	8
11	C6–C7	A	0.5	14	13	1
			Mean	11.45	7.91	3.55
			SD	5.18	4.08	2.15

**Table 4:** Basic characteristics and changeability of hematological parameters measured before and after the exercise in both groups.

Parameter	Test Group			Control Group			Test Group	Control Group	
	Before Exercise	After Exercise	*p*-Value	Before Exercise	After Exercise	*p*-Value	Change After-Before	Change After-Before	*p*-Value
RBC [10^12^·L^−1^]	4.51 ±0.220	4.74 ±0.273	0.0033	5.58 ±0.217	5.77 ±0.363	0.0262	0.23 ±0.161	0.19 ±0.147	1.0000
HCT [%]	40.97 ±2.589	43.47 ±3.229	0.0033	47.06 ±2.226	48.96 ±3.128	0.0164	2.50 ±1.327	1.90 ±0.902	0.4495
Hb [mmol·L^−1^]	8.69 ±0.664	9.12 ±0.758	0.0033	10.07 ±0.529	10.40 ±0.715	0.0185	0.43 ±0.266	0.33 ±0.186	0.5316
PLT [10^9^·L^−1^]	185.00 ±17.378	191.82 ±18.059	0.1095	245.54 ±43.517	285.82 ±57.311	0.0033	6.82 ±11.535	40.27 ±13.794	0.0005
WBC [10^9^·L^−1^]	5.68 ±1.169	6.44 ±0.905	0.0505	5.97 ±0.851	7.81 ±1.221	0.0033	0.76 ±1.002	1.84 ±0.371	0.0233
LYM [%]	38.31 ±7.103	33.72 ±5.786	0.0128	36.11 ±7.150	37.23 ±8.447	0.4498	−4.59 ±5.428	1.12 ±1.297	0.0104
MON [%]	4.34 ±0.835	4.37 ±0.921	0.4069	5.55 ±0.811	5.14 ±0.924	0.0912	0.04 ±0.384	−0.42 ±0.113	0.0659
GRA[%]	58.38 ±7.226	62.58 ±7.226	0.0262	58.34 ±7.370	57.63 ±8.579	0.5937	4.20 ±5.061	−0.70 ±1.209	0.0488

Data are presented as mean ± SD, GRA = granulocyte, Hb = hemoglobin concentration, HCT = hematocrit value, LYM = lymphocytes, MON = monocytes, PLT = thrombocyte count, RBC = red blood cell count, WBC = white blood cell count. Bold indicates the statistically significant values.

## Data Availability

The data presented in this study are available on request from the corresponding author. The data are not publicly available due to the consent provided by participants on the use of confidential data.
